# Altered Accumbal Dopamine Terminal Dynamics Following Chronic Heroin Self-Administration

**DOI:** 10.3390/ijms23158106

**Published:** 2022-07-23

**Authors:** Brianna E. George, Monica H. Dawes, Emily G. Peck, Sara R. Jones

**Affiliations:** Department of Physiology and Pharmacology, Wake Forest School of Medicine, Winston-Salem, NC 27101, USA; bgeorge@wakehealth.edu (B.E.G.); mdawes@wakehealth.edu (M.H.D.); egpeck@wakehealth.edu (E.G.P.)

**Keywords:** heroin self-administration, dopamine, voltammetry, microdialysis

## Abstract

Administration of heroin results in the engagement of multiple brain regions and the rewarding and addictive effects are mediated, at least partially, through activation of the mesolimbic dopamine system. However, less is known about dopamine system function following chronic exposure to heroin. Withdrawal from chronic heroin exposure is likely to drive a state of low dopamine in the nucleus accumbens (NAc), as previously observed during withdrawal from other drug classes. Thus, we aimed to investigate alterations in NAc dopamine terminal function following chronic heroin self-administration to identify a mechanism for dopaminergic adaptations. Adult male Long Evans rats were trained to self-administer heroin (0.05 mg/kg/inf, IV) and then placed on a long access (FR1, 6-h, unlimited inf, 0.05 mg/kg/inf) protocol to induce escalation of intake. Following heroin self-administration, rats had decreased basal extracellular levels of dopamine and blunted dopamine response following a heroin challenge (0.1 mg/kg/inf, IV) in the NAc compared to saline controls. FSCV revealed that heroin-exposed rats exhibited reduced stimulated dopamine release during tonic-like, single-pulse stimulations, but increased phasic-like dopamine release during multi-pulse stimulation trains (5 pulses, 5–100 Hz) in addition to an altered dynamic range of release stimulation intensities when compared to controls. Further, we found that presynaptic D3 autoreceptor and kappa-opioid receptor agonist responsivity were increased following heroin self-administration. These results reveal a marked low dopamine state following heroin exposure and suggest the combination of altered dopamine release dynamics may contribute to increased heroin seeking.

## 1. Introduction

The current opioid overdose epidemic reflects a dramatic increase in the prevalence of opioid use disorder (OUD) and opioid-related overdoses since the 1990s. In 2019, it was estimated that more than 130 people died each day from opioid overdose, with heroin involvement in nearly a third of those overdoses [1]. This epidemic has been exacerbated by the widespread social isolation and social and financial instability experienced during the COVID-19 pandemic, with preliminary reports from the CDC suggesting a 30% increase in opioid-related deaths in 2020 [2]. Furthermore, disruptions in access to clinical providers as a result of COVID-19 restrictions have led to significant reductions in opioid treatment availability and distribution [3,4,5]. There are currently only three medications approved by the FDA to treat OUD: buprenorphine, methadone, and naltrexone. The most effective treatment for OUD is opioid agonist replacement therapy, using either methadone or buprenorphine [6]. However, there are caveats with both medications: methadone has high abuse liability and can cause respiratory depression in patients [7], and buprenorphine is less successful in maintaining patient compliance when compared to methadone-assisted treatment [6]. Therefore, there is a critical need for novel, effective treatments with low abuse potential.

To find potential targets for OUD treatment, it is important to understand the mechanisms of action and consequences of chronic exposure to opioids. The prevailing hypothesis is that the acute reinforcing effects of exogenous opioids occur via disinhibition of dopamine neurons by activation of mu-opioid receptors (MOR) located on GABAergic neurons within the ventral tegmental area (VTA) [8,9,10,11,12]; however, some research has suggested that this may not be the only mechanism involved in opioid reinforcement [13,14,15,16,17]. Support for the disinhibition hypothesis comes from numerous studies that show that opioid-induced disinhibition of VTA dopamine neurons leads to elevation of dopamine levels in the nucleus accumbens (NAc), which is a common mechanism of action across many drugs of abuse.

With regard to the effects of chronic exposure to opioids on dopamine function, there appears to be a general consensus that there is a downregulation of dopamine system function [18,19]. This idea is supported by a large body of evidence that suggests extracellular dopamine and metabolite levels in the striatum (including dorsal and ventral striatum) are decreased during withdrawal following chronic exposure to opioids [20,21,22,23,24]. Furthermore, chronic exposure to opioids causes reductions in VTA dopamine neuron morphology [25,26,27,28]. The firing rate of VTA dopamine neurons was also found to be reduced during opioid withdrawal [29,30]. Changes in dopamine neuron morphology and firing rate last for weeks, well beyond the end of somatic withdrawal signs [25,27,31,32], and may more closely reflect the timeline of withdrawal-related negative affect and reward deficits (for review see [33]).

While substantial evidence exists for opioid-induced adaptations in VTA dopamine neuron function and structure, considerably less is known about the impact of chronic opioid exposure on NAc dopamine terminal function or the regulators of dopamine signaling. Prior studies have shown some changes in dopamine terminals, including changes in dopamine transporter activity and phasic dopamine release [34,35]. In previous studies, our group found that chronic exposure to stimulants or alcohol led to similar reductions in dopamine terminal dynamics and increased responsivity of negative regulators, such as kappa opioid receptors (KOR) and presynaptic D2/D3 autoreceptors [36,37,38,39,40,41]. These receptors are key determinants of overall dopamine levels and increases in function or expression have been found to drive hypodopaminergia and negative affect [18,19,42,43].

Thus, the present study aimed to identify potential mechanisms underlying the cellular and function alterations in dopamine signaling following heroin self-administration. Because hypodopaminergia promotes escalations in drug-seeking and propensity for drug relapse, pinpointing a mechanism for reduced dopamine function following heroin self-administration may lead to potential novel targets for treatments.

## 2. Results

### 2.1. Long access Heroin Self-Administration Leads to Escalation of Heroin Intake

**Figure 1:**
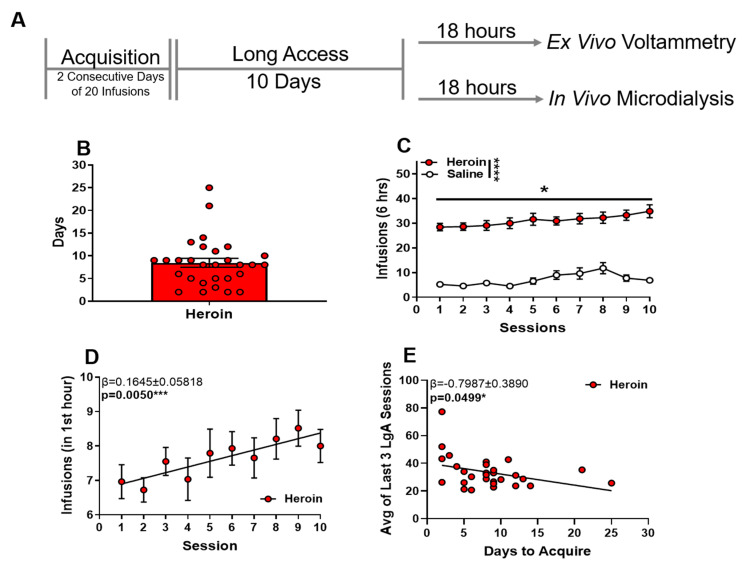
Long access heroin self-administration leads to escalation of heroin intake. (**A**) Experimental timeline of behavioral paradigm. (**B**) The total number of days to reach acquisition criteria. (**C**) Heroin intake during 6-h long access sessions. (**D**) Heroin intake during the first hour of each session. (**E**) Correlation between the number of days to acquisition and average infusions across the last 3 days of heroin long access self-administration. * *p* < 0.05; *** *p* < 0.001; **** *p* < 0.0001.

During acquisition, rats reached acquisition criteria on average in 8.488 ± 0.9821 days (mean ± SEM) (Figure 1B). After acquisition criteria were met, rats were tested for heroin responding (0.05 mg/kg/inf) on an FR1 schedule of reinforcement during six-hour sessions with no limit on rewarded responses. Heroin was self-administered significantly more than saline (Figure 1C). A two-way ANOVA revealed the main effects of the self-administration condition on infusion (Figure 1C, F (1,48) = 131.7, *p* < 0.0001) and session (Figure 1C, F (2.078,99.73) = 4.375, *p* = 0.0140). Heroin infusions within the first hour increased across sessions (Figure 1D, linear regression: β = 1.645 ± 0.05818, *p* = 0.0050), and time to acquisition was negatively correlated with average responding for heroin across the last three sessions (Figure 1E β = −0.07987 ± 0.3890, *p* = 0.0499).

### 2.2. Heroin Self-Administration Reduced Single-Pulse Stimulated Dopamine Release, but Not Dopamine Reuptake in the NAc Core

**Figure 2:**
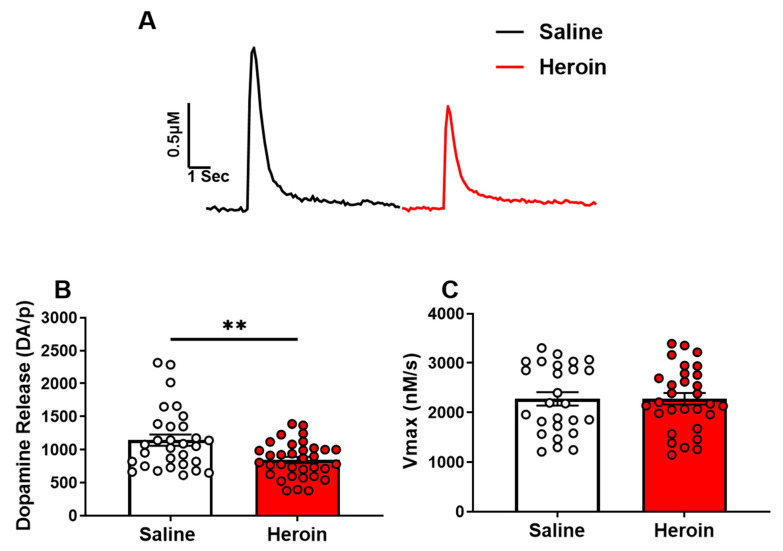
Heroin self-administration reduced single-pulse stimulated dopamine release, but not dopamine reuptake in the NAc core. (**A**) Representative traces of electrically-evoked dopamine in saline exposed (black) and heroin exposed (red) rats. (**B**) Stimulated dopamine release was significantly lower in heroin-exposed rats. (**C**) The maximal rate of dopamine uptake (Vmax) was not significantly different between saline and heroin-exposed rats. ** *p* < 0.01.

Rats were sacrificed for FSCV approximately 18 h following the last heroin self-administration session. We found that single-pulse stimulated dopamine release was significantly lower in heroin-exposed animals (Figure 2B, t_62_ = 3.193, *p* = 0.0022), but that dopamine uptake was not significantly different between heroin-exposed animals and saline controls (Figure 2C, *p* > 0.05).

### 2.3. Opposite Adaptations of D2 and D3 Autoreceptors following Heroin Self-Administration

**Figure 3:**
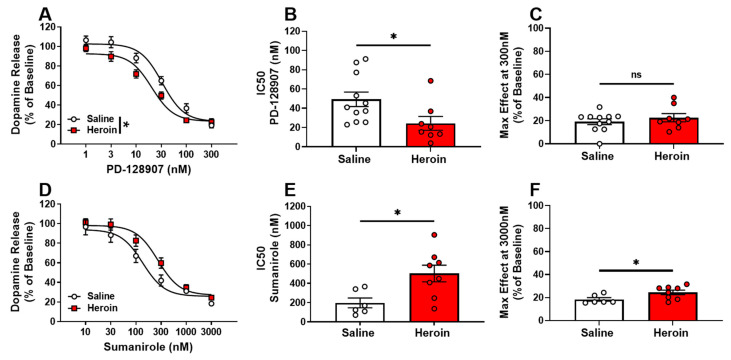
Opposite adaptations of D2 and D3 autoreceptors following heroin self-administration. Concentration-response curves for the selective D3 agonist, PD-128907, and selective D2 agonist, sumanirole, were used to evaluate autoreceptor responsivity. Heroin-exposed animals displayed increased agonist-induced responsivity of D3 autoreceptors (**A**), and a significantly lower IC50 in heroin-exposed animals (**B**), indicating increased agonist potency and no significant differences in PD-128907 maximal effect (at 300 nM), indicating no change in the maximal efficacy of the agonist (**C**). Selective D2 agonist, sumanirole, concentration-response curves (**D**) indicated a significantly greater IC50 (**E**) and maximal effect of sumanirole (**F**) in heroin-exposed rats. * *p* < 0.05; ns: not significant.

To determine if heroin changed the activity of presynaptic D3 autoreceptors and regulation of dopamine release, we examined the release-inhibiting effects of the selective D3 receptor agonist, PD-128907 (Figure 3A). From concentration-response curves, an IC50 of the agonist responsivity and maximal effect of dopamine inhibition at the highest concentration of the curve were calculated from each slice to evaluate the potency and efficacy of agonists, respectively. Heroin self-administration resulted in an increase in D3 autoreceptor responsivity to agonist activation compared to saline controls. Two-way repeated-measures ANOVA revealed a significant main effect of the condition (Figure 3A, F (1,17) = 4.819, *p* = 0.0423), concentration (F (5,85) = 267.3, *p* < 0.0001), and condition × concentration interaction (Figure 3A, F (5,85) = 3.166, *p* = 0.0114). In addition, the IC50 for PD-128907 was significantly decreased in heroin-exposed rats (Figure 3B, t_17_ = 2.317, *p* = 0.0332). This suggests that a lower concentration of agonist was needed to reduce the signal by 50% and indicates a greater agonist potency at D3 autoreceptors in heroin self-administering animals. However, the maximal effect at the highest concentration of PD-128907 (300 nM) was not significantly different between heroin-exposed rats and saline controls (Figure 3C), suggesting no change in the overall efficacy of PD-128907.

To examine changes in D2 autoreceptor-mediated inhibition of dopamine release, the selective D2 receptor agonist, sumanirole, was used (Figure 3D). Similarly, IC50 and the maximal effect of agonist activation were calculated from each concentration-response curve to evaluate the potency and efficacy of the agonist, respectively. Two-way repeated-measures ANOVA revealed a non-significant, but trending, main effect of heroin exposure (Figure 3D, F (1,12) = 3.213, *p* = 0.0983). In addition, the IC50 was significantly increased in heroin-exposed rats (Figure 3E, t_12_ = 2.774, *p* = 0.0168), suggesting reduced potency of the agonist. The maximal effect of the greatest sumanirole concentration (3000 nM) was also increased (Figure 3F, t_12_ = 2.280, *p* = 0.0417), indicating an increase in agonist efficacy.

### 2.4. Presynaptic KOR Activity on NAc Dopamine Terminals Is Increased by Heroin Self-Administration

**Figure 4:**
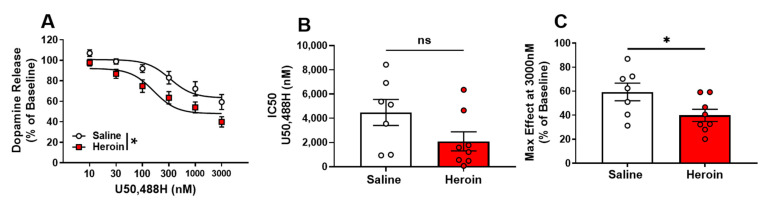
Presynaptic KOR activity on NAc dopamine terminals is increased by heroin self-administration. Selective kappa-opioid receptor agonist U50,488H (**A**) decreased percent baseline dopamine release in heroin exposed rats with (**B**) no significant difference in IC50 between saline exposed and heroin exposed rats, but (**C**) significant decreases in the U50,488H maximal effect in exposed rats. * *p* < 0.05; ns: not significant.

To determine the functional responsivity of KOR following heroin self-administration, the selective KOR agonist, U50,488H (U50), was used. In addition, an IC50 of agonist responsivity and the maximal effect of dopamine inhibition at the highest concentration of the curve were calculated from each slice to evaluate the potency and efficacy of U50, respectively. Concentration response curves of KOR activation by U50 suggest an increased agonist-induced responsivity of KOR in heroin-exposed rats than in saline controls (Figure 4A). A two-way repeated-measures ANOVA revealed a significant main effect of the condition (Figure 4A, F (1,13) = 6.549, *p =* 0.0238), concentration (Figure 4A, F (5,65) = 78.01, *p* < 0.0001), but no significant condition × concentration interaction (Figure 4A, *p* > 0.05). While the IC50 for U50 curves were not significantly different between heroin-exposed rats and saline controls (Figure 4B, *p* = 0.0913), the maximal effect of the highest U50 concentration (3000 nM) was significantly decreased in heroin-exposed rats (Figure 4C, t_13_ = 2.226, *p* = 0.0444). These results indicate that heroin-exposed rats had an increase in U50 efficacy, but no change in U50 potency.

### 2.5. Heroin Self-Administration Alters the Dynamic Range of Dopamine Release

**Figure 5:**
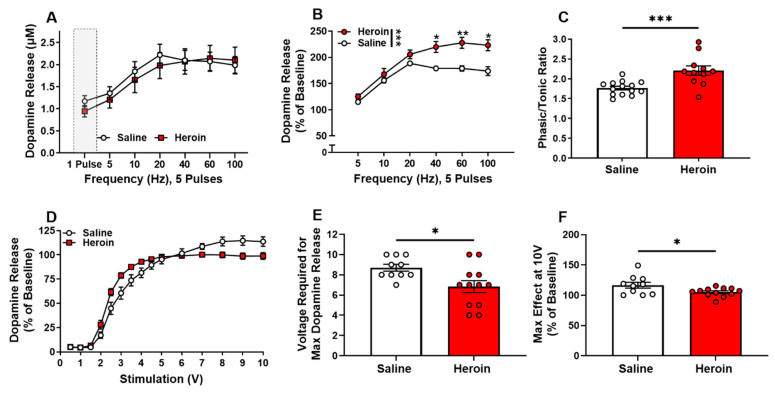
Heroin self-administration alters the dynamic range of dopamine release. Dopamine release was evoked with five pulses at increasing frequencies (5–100 Hz). (**A**) The magnitude of dopamine release did not differ between heroin-exposed and saline animals. (**B**) Percent dopamine release was significantly greater in heroin-exposed rats at high frequencies. (**C**) The multi-pulse phasic/tonic ratio was significantly greater in heroin-exposed rats than in saline control rats. (**D**) Dopamine release following single-pulse electrical stimulation of low and high intensities. Percent dopamine release was significantly greater at low (2.5–4 V) stimulation intensities and was significantly lower at high (7–10 V) stimulation intensities in heroin-exposed rats. (**E**) The stimulation amplitude needed to elicit the greatest dopamine release was reduced in heroin-exposed rats. (**F**) The maximal release by the 10 V stimulation was significantly decreased in heroin-exposed rats. * *p* < 0.05; ** *p* < 0.001; *** *p* < 0.001.

MOR activation results in phasic dopamine firing, which is characterized by a “burst-like” pattern of increased action potentials at higher frequencies [44]. To examine how chronic heroin exposure alters dopamine terminal dynamics, we measured dopamine release under increased stimulation pulses (five pulses) and increasing frequencies (5–100 Hz). When evaluating the absolute magnitude of dopamine release (μM) in response to multi-pulse stimulations, no significant group differences were observed (Figure 5A, *p* > 0.05); however, heroin-exposed rats displayed a frequency-dependent increase in relative phasic release compared to naïve controls when dopamine release was normalized to single-pulse release (Figure 5B). A two-way repeated-measures ANOVA revealed the significant main effects of the condition (Figure 5B, F (1,20) = 15.62, *p* = 0.0008), frequency (Figure 5B, F (1.701,34.02) = 71.27, *p* < 0.0001), and condition × frequency interaction (Figure 5B. F (5100) = 5.551, *p* = 0.001). A Bonferroni’s post-hoc analysis revealed significant differences at 40 Hz (*p* = 0.0224), 60 Hz (*p* = 0.0067) and 100 Hz (*p* = 0.0103). This effect is also reflected in the calculation of phasic/tonic ratio, which compares dopamine release evoked by five pulses at 60 Hz with dopamine release evoked by a single pulse, in heroin-exposed rats (Figure 5C, t_23_ = 3.815, *p* = 0.0009). These results suggest that chronic heroin self-administration may lead to reductions in basal dopamine release while enhancing the signal-to-noise ratio of phasic release events, thereby causing dopamine dynamics to be phasic-shifted. This increase in the gain of dopamine terminal dynamics could lead to increased reinforcement of heroin self-administration and thus drive the increased escalation of intake.

In addition to examining release under “phasic-like” stimulation parameters, we also measured dopamine release by altering the electrical stimulation amplitude. Heroin self-administration increased dopamine release elicited at low stimulation amplitudes and decreased dopamine release elicited by high stimulation intensities (Figure 5D). A two-way repeated-measures ANOVA revealed the significant main effect of the stimulation amplitude (Figure 5D, F (2.121,42.41) = 332.1, *p* < 0.0001) and stimulation amplitude x condition interaction (Figure 5D, F (14,280) = 6.511, *p* < 0.0001). A Bonferroni’s post-hoc analysis did not reveal significant differences at any of the stimulation amplitudes between groups. This effect was quantified by measuring the individual amplitude needed to elicit the greatest amount of dopamine for each slice, which showed that heroin self-administering animals required lower stimulation amplitudes to drive maximal dopamine release across the stimulation intensities tested (Figure 5E, t_20_ = 2.611, *p* = 0.0167). When measuring the amount of dopamine released at the maximal stimulation amplitude (10 V), we found heroin self-administering animals had a lower dopamine release compared to saline controls (Figure 5F, t_20_ = 2.292, *p* = 0.0329). Overall, these results suggest that the dynamic range of dopamine release across varying stimulation intensities is reduced following heroin self-administration.

### 2.6. Heroin Self-Administration Decreased Basal Extracellular Levels of Dopamine in the NAc and Dopamine Response to a Heroin Challenge

**Figure 6:**
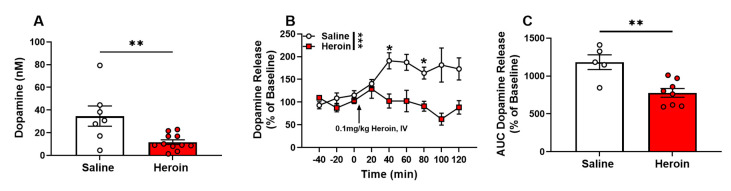
Heroin self-administration decreased basal extracellular levels of dopamine in the NAc and dopamine response to a heroin challenge. (**A**) Overnight (basal) extracellular dopamine was significantly lower in heroin-exposed rats. (**B**) Following the heroin challenge (0.1 mg/kg, IV), heroin-exposed rats showed no significant increase in extracellular dopamine, while significant, prolonged increases were observed in saline rats. (**C**) Heroin-exposed rats showed significantly decreased dopamine when compared to saline control rats. * *p* < 0.05; ** *p* < 0.01; *** *p* < 0.001.

Following one day of recovery after cannula implantation, microdialysis probes were inserted into the NAc, and aCSF was perfused at a flow rate of 0.5 µL/min. A single 15-h baseline sample was collected and analyzed for dopamine at equilibrium between the probe and extracellular tissue, to quantify basal extracellular levels. Approximately 24 to 32 h following the last self-administration session, heroin-exposed animals had a significant decrease in basal levels of extracellular dopamine when compared to saline controls (Figure 6A, t_16_ = 3.052, *p* = 0.0076). The next morning, the flow rate was increased to 1.0 µL/min to allow more rapid (20 min) sampling times. Following the collection of six total samples where dopamine levels were stable (<10% difference and no trend up or down) for at least three collections, all rats were administered 0.1 mg/kg heroin (IV), and samples were collected every 20 min for an additional 120 min. Extracellular dopamine levels were significantly increased in saline control rats following the heroin challenges compared to heroin-exposed rats. A two-way repeated-measures ANOVA revealed the significant main effects of the drug condition (Figure 6B, F (1,12) = 18.79, *p* = 0.0010), time (Figure 6B, F (3.566,42.79) = 2.917, *p* = 0.0370) and condition x time interaction (Figure 6B, F (8,96) = 4.939, *p* < 0.0001). A Bonferroni’s post-hoc analysis revealed significant differences at the 40 (*p* = 0.0293) and 80 (*p* = 0.0128) minute time points. Analysis of the area under the curve for the magnitude of dopamine outflow showed dopamine release was significantly lower in heroin-exposed animals than in saline controls (Figure 6C, t_11_ = 3.840, *p* = 0.0027).

## 3. Discussion

The current study demonstrated that a history of chronic, long-access heroin self-administration in rats induced adaptations in the extracellular levels, terminal release dynamics, and presynaptic regulators of the dopamine system that are consistent with an overall state of hypodopaminergia. While there is evidence to support alterations in VTA dopamine neuron firing and morphology [29,37,38,45,46,47], it is not fully known if this reflects the functional mechanism underlying reduced accumbal dopamine levels. In combination, this evidence suggests that chronic opioid exposure and withdrawal results in marked adaptations and alterations in dopaminergic functioning. Understanding the cellular mechanisms underlying heroin-induced hypodopaminergia will be important in combating OUD, since low dopamine function is strongly associated with an increased propensity to seek and take drugs and an increased probability of relapse after abstinence [48,49,50].

When given access to heroin under a long access procedure, male rats escalated their intake across sessions, as shown previously [51,52,53,54]. The literature shows that rodent heroin self-administration protocols lead to tolerance and drug withdrawal symptomology, such as body shakes, diarrhea, writhing, and teeth chatter that are similar to withdrawal symptoms observed in clinical populations with OUD [45,46,55]. Therefore, we suggest that heroin self-administration is a useful model to evaluate heroin-induced alterations in neurobiology.

In this study, heroin self-administering rats displayed reduced basal extracellular levels of dopamine and reduced dopamine release in response to single-pulse electrical stimulations in the NAc, which are considered classical signs of reduced dopamine system function, or hypodopaminergia [47]. The present findings are in agreement with previous studies showing that chronic exposure to opioids leads to reduced extracellular levels of dopamine in the striatum [24,56,57]. Furthermore, we found that an acute IV heroin challenge, which normally increases the extracellular levels of dopamine in naïve rats, had no effect in self-administering animals. This tolerance-like effect may provide a neurobiological basis for the consistent self-reports from OUD sufferers indicating that, after months or years of daily use, heroin no longer makes them “high” [58,59]. Combined, these results suggest that chronic heroin exposure, like exposure to other drugs of abuse, leads to tolerance and hypodopaminergia. Hypodopaminergia, along with augmented neurobiological stress responses, has been linked to compensatory increases in drug-seeking behaviors and negative affect during drug withdrawal [18]. Thus, alterations in the downregulations in dopamine functions observed following chronic heroin self-administration may contribute to the escalation of seeking seen during long access as a way to alleviate withdrawal-related negative affect.

Here, we report that, after extended heroin self-administration, D2 autoreceptors exhibit a decrease in agonist-induced responsivity, while D3 autoreceptors exhibit increased responsivity. To our knowledge, this is the first report that provides evidence of a functional increase in D3 autoreceptor activity in the NAc after opioid self-administration. D2-type (D2 and D3) autoreceptors modulate the release, synthesis, and the firing rate of dopamine neurons, and are expressed on the soma, dendrites, and axon terminals of dopamine neurons [60,61]. While there is a large body of literature supporting the role of D2 autoreceptors in dopamine feedback regulation, the evidence for D3 receptors acting as autoreceptors is contradictory [62,63]. The variability in the evidence to support D3Rs as autoreceptors may be due to technical limitations, given the structural similarities between D2 and D3 receptors (which limits the specificity of ligands), and the 10-fold greater density of D2 receptors in the striatum [64,65]. While acknowledging that most D2R and D3R selective agonists, including the ones used in this report, will act on both receptors at high concentrations, there were nonetheless clear and opposite shifts in the concentration-response curves of these drugs after heroin self-administration, indicating decreased responsiveness of D2 autoreceptors alongside increased responsiveness of D3 autoreceptors. This strongly suggests a functional dichotomy between the two receptors.

In this study, we isolated the effects of D2 or D3 autoreceptors and excluded postsynaptic receptors by evoking dopamine release with electrical stimulation in nucleus accumbens slices and measuring the release-inhibitory effects of D2- or D3-preferring ligands. With the lower density [64] but greater sensitivity of D3Rs for dopamine relative to D2Rs [66], it is difficult to predict the net effect of the opposite changes between these receptors. Interestingly, it has been hypothesized that, due to their high affinity for dopamine and localization on asymmetric synapses associated with volume transmission, these receptors are likely to be less sensitive to rapid, phasic release events than to slower, tonic changes in the basal dopamine level [67]. Thus, increased D3 autoreceptor function may lower basal levels, reduce D2 autoreceptor-mediated inhibition and increase the size and signal-to-noise ratio of phasic dopamine release, potentially leading to a state of aberrantly high saliency of external stimuli such as drug cues.

Other studies have reported opposing D2R and D3R changes in mesolimbic regions following chronic exposure to opioids in rodents [68,69,70,71] and humans [72,73], with D2R binding being downregulated and D3R binding being upregulated. However, there have been limited attempts to differentiate D2 and D3 autoreceptors from postsynaptic receptors, and given the greater expression of postsynaptic D2R [74,75], the prior results may better reflect postsynaptic receptor changes. The results of this autoreceptor study are intriguing in light of other studies, which show that D3R antagonists are effective at reducing opioid self-administration [76,77,78,79,80,81,82] (for review see [83,84,85,86]). D3R antagonists may likely work at both the presynaptic receptors to induce an increased basal dopamine tone and alleviate withdrawal-induced hypodopaminergia, while simultaneously blocking the postsynaptic receptors, thus reducing synaptic dopamine transmission. Combined, these effects would reduce opioid seeking by alleviating negative affect during withdrawal in addition to reducing dopamine signaling in the presence of opioids [87]. Thus, more research evaluating the signaling and functional properties of the D3 autoreceptor is highly warranted.

Similar to the D3 autoreceptors, there was also increased activity of another inhibitory presynaptic dopamine regulator, the dynorphin/KOR system, following heroin self-administration. Previous studies show that KORs are located on presynaptic dopamine terminals and, upon activation with their endogenous ligand, dynorphin, strongly inhibit dopamine release [41]. Prior studies show that dynorphin and its precursors are elevated following opioid exposure [88,89,90,91,92], suggesting that endogenous activity of KORs may be increased. Furthermore, administration of the KOR antagonist, norbinaltorphimine (nor-BNI), is effective at reducing heroin self-administration and withdrawal-induced negative affect [93]. In addition, another study reported that co-administration of heroin and the KOR agonist, U50,488H, reduced heroin-induced NAc dopamine release during self-administration, and low doses of U50 increased heroin self-administration [94]. Combined, these data suggest that inhibition of dopamine release through endogenous activation of the KOR as a result of chronic exposure may decrease the reinforcing properties of heroin and promote negative affect, thereby driving increased opioid seeking, particularly during withdrawal. The results of the current study expand upon previous findings, showing that KOR activity is increased after chronic opioid administration, and providing further evidence that a KOR antagonist may be a viable treatment option for OUD. While the alterations in KOR and D2/D3 autoreceptor function provide initial evidence for a functional change, future studies should assess if this functional change is due to a change in the expression or signaling capabilities of these receptors.

Perhaps the most surprising discovery was that dopamine release under “phasic-like” electrical stimulation was increased in the NAc following heroin self-administration while tonic release was reduced. However, while heroin-exposed rats exhibited greater release than the control group at low amplitude stimulations, at high amplitudes, there was reduced release. A previous study showed that MOR activation results in presynaptic facilitation in the NAc, likely through inhibition of cholinergic interneuron activity as seen with nicotinic antagonists [95]. This presynaptic facilitation results in reduced dopamine release under single-pulse stimulations and subsequent enhancement of dopamine release under burst-like stimulations due to increased accumulation of presynaptic calcium [96,97]. Thus, it is possible that the mechanisms by which nicotine and opioids regulate dopamine release in the NAc have some functional overlap [95].

This idea is further supported by evidence showing that chronic nicotine exposure leads to reduced single-pulse dopamine release, increased paired-pulse ratios, and increased phasic/tonic ratios in the NAc [98,99]. This is consistent with our findings as, when normalized to single-pulse release, we observed increased dopamine release under multi-pulse stimulations in heroin-exposed animals compared to saline controls; however, the absolute magnitude of dopamine release under single-pulse stimulations was reduced in heroin-exposed animals compared to their saline counterparts. This finding is enhanced by results from the stimulation intensity experiment, which show that dopamine release was facilitated at low stimulation intensities and decreased during high stimulation intensities during single-pulse stimulations. While this finding may seem counterintuitive, this may suggest that the dynamic range of dopamine release is largely reduced following chronic heroin exposure. In a similar manner to the previous experiment, while lower stimulation intensities increased release when normalized to their single-pulse baseline release in heroin-exposed animals due to reduced basal levels of dopamine, the overall magnitude of dopamine release is still reduced in heroin-exposed animals compared to their naïve counterparts. This result coupled with the finding that dopamine release is reduced under high stimulation intensities in heroin-exposed rats suggests a restricted range of activity-dependent dopamine release, which has been previously shown to occur following chronic nicotine exposure and is thought to contribute to altered coding of dopamine reward signaling [100].

Our group recently showed that extended heroin self-administration does not lead to increased dopamine release under “burst-like” stimulations in male rats [34]. While these previous results may appear contradictory when compared to the current findings, some methodological differences may account for the differing results. In the previous study, dopamine recordings were conducted in the medial NAc shell, while the current study was in the NAc core. The NAc shell is known to have a different dopaminergic innervation [97,101] and distribution of dopamine regulators, such as autoreceptors, KOR, and DAT, as compared to the NAc core [102,103], suggesting that variances in the regulation of dopamine transmission may account for some of the observed differences [104]. Second, in the prior study, a lower dose of heroin (0.025 mg/kg/inf) was used compared to the current study (0.05 mg/kg/inf). Therefore, it is possible that the lower dose was not sufficient to elicit the adaptions to phasic dopamine release seen in this experiment.

Overall, this study builds support for a mechanism in which chronic exposure to heroin results in reduced dopamine system function and highlights potential targets for pharmacological interventions, including presynaptic D3 receptors and KORs. Therapeutics aimed at restoring dopamine levels after chronic opioid exposure may be beneficial in treating OUD. While the current study measured outcomes during what would commonly be considered an “early withdrawal” time point, it would be interesting to evaluate if these alterations in dopamine function persist or are even exacerbated during late withdrawal.

## 4. Methods and Materials

### 4.1. Animals

Adult male Long Evans rats (300–400 g, Envigo, Indianapolis, IN, USA) were given ad libitum access to food and water and maintained on a 12-h modified reversed light/dark cycle (0300 h lights off; 1500 h lights on). Animal care procedures were in accordance with the National Institutes of Health guidelines in the Association for Assessment and Accreditation of Laboratory Animal Care and the experimental protocol was approved by the Institutional Animal Care and Use Committee at Wake Forest School of Medicine.

### 4.2. Intravenous Heroin Self-Administration

Intravenous catheter surgery: Rats (Heroin: *n* = 29; Saline: *n* = 21) were anesthetized with ketamine (100 mg/kg, i.p.) and xylazine (10 mg/kg, i.p.) and implanted with a chronic indwelling jugular catheter as described previously [34,105]. Immediately following the surgery, rats were administered meloxicam (1 mg/kg, s.c.) as a postsurgical anesthetic and singly housed in custom chambers that functioned as both a housing cage and operant chamber.

Heroin and Saline Self-Administration Procedures: Self-administration sessions were conducted during the active/dark cycle, between 0900 and 1500 h. After at least two days of recovery, rats were trained to lever press for heroin on a fixed ratio one (FR1) schedule of reinforcement using a single active lever which, when pressed, delivered an intravenous infusion of heroin (0.05 mg/kg/inf). Acquisition self-administration sessions were initiated with the extension of the active lever into the chamber, with no experimenter-delivered priming infusions, and were terminated after 20 infusions or 6 hours, whichever occurred first. During the sessions, each lever response resulted in a 20-s time-out period in which the cue light was illuminated. Acquisition criteria were set as two consecutive daily sessions during which 20 infusions were obtained. When the acquisition criteria were met, rats were placed on a long access paradigm with unlimited access to heroin (0.05 mg/kg/inf) on an FR1 schedule of reinforcement during 6-hour sessions occurring across 10 consecutive days. A separate group of age-matched rats was given access to a saline-paired lever and allowed to self-administer saline under the same parameters of long access. Saline self-administration rats received approximately 20 sessions to match the sessions needed to complete acquisition and long access for heroin self-administering rats.

### 4.3. Ex Vivo Fast-Scan Cyclic Voltammetry

Fast-scan cyclic voltammetry (FSCV) was used to examine the presynaptic NAc dopamine terminal release kinetics and dopamine autoreceptor and kappa opioid receptor (KOR) activity. Approximately 18 h following the last long access session, rats were deeply anesthetized with isoflurane and rapidly decapitated. The brain was removed and immersed in oxygenated artificial cerebrospinal fluid (aCSF) containing (in mM): NaCl (126), KCl (2.5), NaH_2_PO_4_ (1.2), CaCl_2_ (2.4), MgCl_2_ (1.2), NaHCO_3_ (25), glucose (11), L-ascorbic acid (0.4). A vibrating tissue slicer (Leica VT1200S, Leica Biosystems, Wetzler, Germany) was used to prepare 400 µm thick coronal brain slices containing the NAc core. Slices were transferred to a recording chamber and submerged in a bath of oxygenated aCSF (32 °C) perfused at a rate of 1 mL/min. A carbon-fiber microelectrode (CFE; 100–200 μM length, 7 μM radius) and bipolar stimulating electrode were placed in the NAc core. Endogenous dopamine release was electrically evoked by a single pulse (7.5 V, 4 msec, monophasic) applied to the slice every 3 min. Extracellular dopamine was measured by applying a triangular waveform (−0.4 to + 1.2 to −0.4 V vs. Ag/AgCl, 400 V/s) to the CFE. After an hour of slice acclimation and when the dopamine signal was stable for at least three successive collections, slices were used for one of the following experiments listed below.

D2/D3 Autoreceptor and KOR Activity: To probe the responsivity of presynaptic D3 autoreceptors in the regulation of terminal release, cumulative concentrations of the D3 agonist, PD-128907 (1–300 nM; half-log steps), were bath applied to separate slices (Heroin *n* = 8 rats/slices; Saline *n* = 11 rats/slices). The responsivity of presynaptic D2 autoreceptors was evaluated using the D2 agonist, sumanirole (1–3000 nM; half-log steps) (Heroin *n* = 8 rats/slices; Saline *n* = 6 rats/slices). KOR activity was measured using the KOR agonist, U50-488H (1–3000 nM) (Heroin *n* = 8 rats/slices; Saline *n* = 7 rats/slices). Each slice was exposed to only one drug. The interstimulus interval for all concentration curves was 3 min.

DA Release across Varying Stimulation Parameters: The effects of two different stimulation patterns on dopamine release were tested. A frequency-response curve was obtained using five pulse stimulations at 5, 10, 20, 40, 60, and 100 Hz frequencies with 5-min interstimulus intervals (Heroin and Saline: *n* = 4 rats each, 10–12 slices total). A stimulation intensity curve was collected by using a single pulse stimulation at varying intensities, 50, 100, 150, 200, 250, 300, 350, 400, 450, 500, 600, 700, 800, 900, 1000 V with an interstimulus interval of 60 s (Heroin and Saline: *n* = 4 rats each, 10–12 slices total). From this dataset, the stimulation intensity needed to elicit the greatest dopamine response and the dopamine change elicited at the highest stimulation intensity (10 V) was used to quantify these results.

FSCV Data Analysis: Demon Voltammetry and Analysis software [106] was used to analyze all FSCV data. Recording electrodes were calibrated at the end of every experiment by washing a known concentration of dopamine over the CFE using a flow-injection system and measuring the resulting current. The calibration factor of each electrode was used to convert the electrical current measured during experiments to dopamine concentration. Michaelis–Menten modeling was used to determine the concentration of dopamine released and the maximal rate of uptake (V_max_) following electrical stimulation.

### 4.4. In Vivo Microdialysis

Microdialysis surgeries were performed approximately 18 h following the final long access self-administration session (Heroin *n* = 5; Saline *n* = 5). Guide cannula (MD-2250; BASi Instruments, West Lafayette, IN, USA) were stereotaxically implanted 2 mm above the NAc (AP: +1.6 mm; L: ± 1.4 mm; DV: −6.0 mm), as the probe extends 2 mm past the cannula. Concentric microdialysis probes (2 mm membrane, MD-2200; BASi Instruments, West Lafayette, IN, USA) were inserted approximately 36 h post-surgery and continuously perfused with artificial cerebrospinal fluid (aCSF; pH 7.4; NaCl 148 mM, KCl 2.7 mM, CaCl_2_ 1.2 mM, MgCl_2_ 0.85 mM). For basal extracellular dopamine level experiments, overnight samples were collected at 0.5 µL/min from 1700 h to 0800 h, at which time the flow rate was increased to 1.0 µL/min. Baseline samples were collected every 20 min for 2 hours before intravenous administration of 0.1 mg/kg heroin. Following administration of heroin, 20-min duration samples were collected for another 2 hours. All dialysate samples were frozen at −80° before the determination of dopamine concentrations by high-performance liquid chromatography (HPLC) with electrochemical detection (ESA/Thermo Scientific, Chelmsford, MA, USA)

High-Performance Liquid Chromatography. All dialysate samples were analyzed using high-performance liquid chromography (HPLC) (ESA/Thermo Scientific, Chelmsford, MA, USA) coupled with electrochemical detection at +220 mV using a high sensitivity analytical cell 5011 A (Thermo Fisher Scientific, Sunnyvale, CA) on a Choulechem III Electrochemical Detector (ESA/Thermo Scientific, Chelmsford, MA, USA). Neurotransmitters and their metabolites were separated on a Luna 100 × 3.0 mm C18 3 μm HPLC reversed-phase column (Phenomenex, Torrance, CA). The mobile phase consisted of 75 mM NaH2PO4, 1.7 mM 1-octanesulfonic acid sodium salt, 100 µL/L triethylamine, 25 µM EDTA, 10% acetonitrile *v/v*, at pH = 3.0. Analytes were quantified using Chromeleon software (ESA/Thermo Scientific, Chelmsford, MA, USA) by standards with known dopamine concentrations.

### 4.5. Statistical Analyses

GraphPad Prism 9 (La Jolla, CA, USA) was used to statistically analyze all datasets and create graphs. Behavioral data were analyzed using two-way repeated-measures ANOVA followed by Bonferroni’s post-hoc test where noted. Pearson’s correlation tests were used to measure the strength of the correlations. Baseline voltammetry release and reuptake data, IC50, maximal effect, phasic/tonic ratio, microdialysis basal, and area under the curve data were compared using a Student’s *t*-test. Voltammetry concentration-response curves, multi-pulse release curves, and microdialysis data were subjected to a two-way ANOVA, with group differences being tested using Bonferroni’s post-hoc tests where noted. All data are reported as mean ± SEM. All *p*-values of <0.05 were considered to be statistically significant.

## Data Availability

The data presented in this study are available on request from the corresponding author.
